# A Systematic Review on Ketamine and Esketamine for Treatment-Resistant Depression and Suicidality in Adolescents: A New Hope?

**DOI:** 10.3390/children11070801

**Published:** 2024-06-29

**Authors:** Simone Pardossi, Andrea Fagiolini, Simona Scheggi, Alessandro Cuomo

**Affiliations:** Department of Molecular Medicine, University of Siena School of Medicine, 53100 Siena, Italy; andrea.fagiolini@unisi.it (A.F.); simona.scheggi@unisi.it (S.S.); alessandro.cuomo@unisi.it (A.C.)

**Keywords:** treatment-resistant depression, adolescent depression, ketamine, esketamine, suicidal ideation, suicide

## Abstract

Treating depression in adolescents is a significant challenge, and major depressive disorder (MDD) with suicidal ideation and treatment-resistant depression (TRD) are common and potentially devastating to optimal psychological and physical development in this age group. Suicide is among the leading causes of youth mortality, and TRD occurs in up to 40% of adolescents with MDD. TRD involves severe, persistent symptoms that are hard to treat, significantly reducing functioning and quality of life. We conducted a literature search focusing on key terms related to ketamine and esketamine for MDD with suicidal ideation and TRD in adolescents, aiming to review the potential utility of these molecules in adolescents for these conditions. Ketamine has shown efficacy in reducing depressive symptoms in adolescents with TRD. Esketamine has shown efficacy in reducing depressive symptoms and treating suicidal ideation in adolescents. Both ketamine and esketamine have demonstrated favorable safety and tolerability profiles. Using these drugs for serious conditions like adolescent MDD with suicidal thoughts and TRD can effectively treat symptoms, reduce self-harm and suicide risks, and provide a window for longer-term therapeutic interventions. The prompt and effective treatment of TRD could improve adolescents’ quality of life. However, more research is needed to optimize treatment protocols and evaluate long-term effects.

## 1. Introduction

Adolescent depression represents a significant mental health challenge, with symptoms that can predict major depressive episodes in adulthood, even among adolescents without a major depressive disorder (MDD) diagnosis [1]. A systematic review and meta-analysis found that the prevalence of MDD among adolescents was 8%, and, for dysthymia, it was 4% [2]. Another study indicated that approximately 10–15% of all children and adolescents are currently experiencing depressive symptoms, with 2% of young children and 4–8% of adolescents suffering from MDD [3]. Risk factors associated with depression in adolescence include a family history of depression, female gender, the presence of subthreshold depressive symptoms, non-affective disorders, negative cognitions, interpersonal conflicts, poor social support networks, and stressful events [4,5].

Furthermore, adolescent depression significantly increases the risk of suicide, which is the second leading cause of death among adolescents [6,7,8]. Several studies have highlighted that mental disorders and substance abuse are the most important risk factors for both suicide attempts and completed suicides in adolescents [6,7,8]. Specifically, the presence of MDD has consistently been identified as a key risk factor for suicide, suicidal ideation, and/or suicide attempts [7,9]. According to the Clinical Report of the American Academy of Pediatrics [10] on suicide and suicide risk in adolescents, treating the underlying disorders contributing to suicidal behavior is essential, whether through psychotherapy, psychopharmacotherapy, or a combination of both. Selective serotonin reuptake inhibitors (SSRIs) are the first-line pharmacologic treatment for depression and anxiety, shown to reduce suicidal ideation in adults over 25. The Food and Drug Administration (FDA)’s 2004 black box warning on antidepressants for those under 25, although debated [11], highlights the need to closely watch and control the risk of suicidal thoughts and behaviors, underscoring the necessity for vigilant oversight and the consideration of other safer, more effective, and rapid options.

Clinical guidelines for the acute management of adolescent depression recommend the use of SSRIs, psychotherapy, or both, with cognitive behavioral therapy (CBT) being the most studied form [12]. SSRIs are the most commonly used antidepressants for treating depression in adolescents. The FDA has approved two SSRIs for this purpose: fluoxetine, which is approved for children aged 8 and older [13], and escitalopram, approved for those aged 12 and older [14]. The European Medicines Agency (EMA) has similarly approved fluoxetine for use in children and adolescents aged 8 years and older for the treatment of MDD [15]. For escitalopram, the EMA has approved it for adolescents aged 12 and older for the treatment of MDD [16]. Despite these treatments’ efficacy, about 40% of adolescents do not respond adequately [17,18] leading to the classification of a subgroup known as “treatment-resistant depression (TRD) in adolescents” [19]. Although there is currently no universally accepted operational definition of TRD [20], the most used definition requires the failure of two or more antidepressant therapies, considering dose and duration adjustments. TRD is associated with a high recurrence of depression and an increased risk of other psychopathological disorders, such as MDD, anxiety disorders, and suicide [20]. Clinical predictors of poor outcomes in the acute treatment of adolescent depression include the chronicity and severity of depression, nonsuicidal self-injury, suicidal ideation, and hopelessness [17,18,19,21,22,23]. Additionally, comorbid anxiety and depressive symptoms in children and adolescents are associated with a greater symptom severity and treatment resistance compared to those with either condition in isolation [24]. Untreated adolescent depression can have long-term impacts, with adolescents suffering from MDD being more likely to develop depression and other mental disorders in adulthood. Even subthreshold depression is associated with an increased risk of future MDD [1,18].

The management of TRD in adolescents presents a significant challenge despite the availability of various treatment strategies. The Treatment of Resistant Depression in Adolescents (TORDIA) trial, for example, classified adolescents as resistant if their depressive symptoms persisted despite at least eight weeks of SSRI treatment, with the last four weeks at a dosage equivalent to at least 40 mg of fluoxetine. This indicates that SSRIs alone may not always be sufficient in these cases. The TORDIA trial demonstrated that combining medication switches with CBT can effectively improve outcomes for adolescents with TRD [17,25]. Pharmacotherapy strategies may include switching medications to other SSRIs or SNRIs, like venlafaxine [26]. Transcranial magnetic stimulation (TMS) has also been considered as a treatment option [27]. Psychotherapy, particularly CBT, is highly effective, especially when combined with medication, as demonstrated by the TORDIA trial [25].

The literature contains limited evidence on the treatment and management of adolescent treatment-resistant depression, making it a condition that often remains difficult to resolve [19]. Despite advancements in treatment modalities and interventions, the management of MDD with suicide ideation and TRD in adolescents continues to present significant clinical and therapeutic challenges. Given these ongoing difficulties, there is growing interest in exploring innovative treatments such as ketamine and esketamine. These agents have shown promise in rapidly alleviating depressive symptoms, offering a potential new avenue for those who have not responded to traditional therapies [22,23,24,25,26,27]. Ketamine inhibits GABA interneurons, resulting in the disinhibition of pyramidal neurons, increased glutamate release, and activation of AMPA receptors [28,29]. Ketamine also promotes neurogenesis by activating AMPA receptors and increasing BDNF expression, which stimulates pathways like PI3K/Akt and MEK-MAPK/Erk, activating mTOR, a crucial regulator of neurogenesis and synaptic growth [30,31]. The antidepressant effects of ketamine are diminished with rapamycin, an mTOR inhibitor [32]. Additionally, ketamine reduces hyperactivity in the lateral habenula (LHb) [33]. It also affects extra-synaptic NMDA receptors, preventing the chronic suppression of protein synthesis and enhancing synaptic potentiation [30,34]. Furthermore, ketamine interacts with μ and κ opioid receptors [35]. Beyond NMDA receptor antagonism, ketamine influences monoaminergic systems by inhibiting serotonin, norepinephrine, and dopamine transport, and interacting with GABAa receptors, which contributes to its anxiolytic effects [36,37].

In adolescents, ketamine’s neurobiological effects have been studied with a focus on brain entropy and neurotrophic molecular markers [38,39]. It was found that ketamine treatment increased brain entropy, suggesting enhanced neuroplasticity and brain function adaptability [38]. Additionally, a significant rise in BDNF levels post-treatment indicated the promotion of neuronal growth and synaptic plasticity, correlating with clinical improvements in depressive symptoms [38]. In another study, fMRI was used to observe changes in brain activity in adolescents with treatment-resistant depression before and after ketamine treatment [39]. Significant alterations were noted in the prefrontal cortex and amygdala, correlating with reductions in depressive symptoms and improvements in cognitive functions and overall behavior [39]. Clinically, it is crucial that we address adolescent depression promptly not only to alleviate suffering and improve quality of life but also because untreated depression significantly increases the risk of suicide among this age group. Studies have shown that depression is a major risk factor for suicidal ideation and attempts in adolescents [7,8]. The approval of esketamine, the S-enantiomer of ketamine, by major regulatory authorities such as the FDA and EMA for TRD has indeed been a significant advancement in the treatment of TRD. Several studies have demonstrated esketamine efficacy and safety in adults, even in the long term [40,41,42,43,44,45]. Other reviews have investigated the general efficacy of ketamine in treating adolescent and pediatric depression, predominantly focusing on the use of intravenous ketamine at varying doses, highlighting its potential as a rapidly acting antidepressant [46,47]. A recent review investigated the role of ketamine for mood disorders, anxiety, and suicidality in children and adolescents, highlighting that ketamine may be safe and effective for these conditions in youth [48].

The objective of our study is to review the existing literature on the use of ketamine and esketamine in TRD and MDD with suicide thoughts among adolescents, extrapolating the available information on their efficacy, safety, and tolerability.

## 2. Materials and Methods

**Figure 1:**
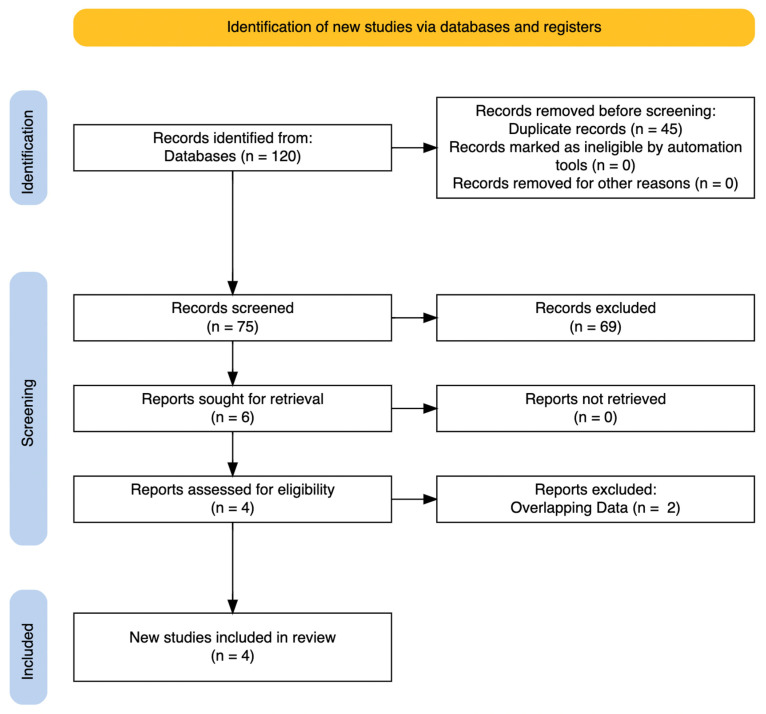
PRISMA flow-chart for selection and inclusion of studies.

We followed the PRISMA guidelines for this systematic review [49] (Figure 1). Our search was conducted across multiple databases: MEDLINE, Scopus, and Cochrane Central Register of Controlled Trials were screened, using keywords related to ketamine, esketamine, adolescents, treatment-resistant depression, and depression with suicidal ideation We reviewed the published literature up to the end of May 2024. We performed the research in June 2024. Additionally, an independent search was performed in Google Scholar to identify any papers that might have been missed. Conference abstracts were reviewed, but none contained sufficient data for inclusion. Hand searches were performed independently by two calibrated investigators (S.P. and A.C.) in relevant journals. Selection was performed by two independent and calibrated reviewers (S.P. and A.C.). Any disagreement at this stage was resolved under the supervision of A.F.

As summarized in Figure 1, we initially eliminated duplicates. We included randomized controlled trials (RCTs) and studies that examined the efficacy of ketamine and/or esketamine, in any formulation, for adolescents diagnosed with TRD or MDD with suicidal ideation, considering populations aged 12 to 18 years. To comprehensively integrate the existing literature on the topic, we also considered appropriate registered trials and open-label studies. We excluded studies that did not address the treatment of depressive symptoms with ketamine or esketamine, studies using animal models, studies lacking sufficient data on changes in depressive symptoms with treatment, case reports, and case studies. Data collection was carried using a structured extraction sheet during the full-text analysis. Study characteristics included: country, number of participants, age, diagnosis definition, study duration, clinical ratings, severity of symptoms, and side effects. Authors were contacted for clarification of information when necessary.

Data collection was performed by two independent reviewers (S.P. and A.C.). The primary outcome of interest was the score of depressive symptoms, as measured by the Montgomery–Åsberg Depression Rating Scale (MADRS) or the Children’s Depression Rating Scale—Revised (CDRS-R). Regarding suicidal ideation, we used specific outcome scales such as the Columbia Suicide Severity Rating Scale (C-SSRS). We also considered other scales that measure various aspects of depressive symptoms, whenever they could be accessed. Authors were contacted when information was unclear at this stage.

For the outcomes in the RCTs, we used the mean difference along with the 95% confidence intervals (CI) as the effect measure. Specifically, we calculated the mean differences between the groups and provided the confidence intervals to indicate the precision of these estimates. For the open-label studies, we reported the improvement in the respective scales over time, without calculating a specific effect measure. This included documenting changes in the scores to illustrate the treatment’s impact on the measured outcomes over the duration of the study.

The overall quality of the included clinical trials, including RCTs and open-label studies, was evaluated using appropriate tools for each type of study. For the RCTs, we used the Cochrane Risk of Bias 2.0 (RoB2) tool [50] to assess risk of bias across several domains: randomization process, deviations from intended interventions, missing outcome data, measurement of the outcome, and selection of the reported outcome. Each domain was rated as “low risk”, “some concerns”, or “high risk”. For the open-label study, we employed the ROBINS-I tool (Risk Of Bias In Non-randomized Studies—of Interventions) [51] to evaluate risk of bias. This tool assesses bias in domains such as confounding, selection of participants, classification of interventions, deviations from intended interventions, missing data, measurement of outcomes, and selection of the reported result. Each domain was rated as “low risk”, “moderate risk”, “serious risk”, or “critical risk”, with an overall risk of bias score determined. Risk of bias visualization (robvis) tool [52] was used to create the figures (Supplementary Materials: Figures S1 and S2). Risk of bias was independently assessed by two reviewers (S.P. and A.C.). Any discrepancies between the reviewers were resolved through discussion. In cases where consensus could not be reached, a third reviewer (A.F.) was consulted. The inter-rater agreement was quantified using the kappa statistic. The overall quality of evidence at the outcome level was evaluated using the GRADE approach (Supplementary Materials: Table S1) [53]. The quality was rated on a four-point scale (very low, low, moderate, and high) based on factors such as study design, risk of bias, inconsistency, indirectness, and imprecision, with each factor rated as very serious, serious, or not serious [53]. Consequently, the strength of the recommendation was categorized as critical, important, or not important [54].

## 3. Results

The systematic electronic search yielded a total of 120 records, which reduced to 75 after removing duplicates. An initial eligibility screening based on article titles and abstracts led to the exclusion of 69 articles: animal model studies, studies on individuals not aged between 12 and 18 years, studies with insufficient data regarding depressive symptoms or symptoms related to suicidal ideation, and non-English studies were excluded. Case studies and case reports were not included in the systematic review, although some are cited in the discussion for their potential clinical utility. We included two RCTs, a registered clinical trial and an open-label study in our review. Six articles underwent a thorough full-text review, four of which were included in the further analysis. While all six studies were valid, two [55,56] were excluded because they contained overlapping results with the third study [57].

The characteristics of the selected studies are summarized in Table 1. Three studies were conducted in the USA [58,59,60], and one was conducted in China [57]. Three studies are RCTs [57,58,60], one of which is a crossover design [58], while one study is an open-label study [59]. Two studies included adolescents with MDD and suicidal ideation [57,60], while the other two included adolescents with TRD [58,59]. Two studies utilized intravenous ketamine [58,59], one used intravenous esketamine [57], and one used intranasal esketamine [60]. All studies measured depressive symptoms and their variations using psychometric scales such as the MADRS and the CDRS-R for depressive symptoms, and the C-SSRS [57] for suicidal ideation.

Zhou’s study [57] highlights the rapid and significant reduction in depressive symptoms and suicidal ideation scores following intravenous esketamine treatment. Specifically, the study involved 54 participants aged 13–18 with MDD and suicidal ideation, who received three infusions of either esketamine (0.25 mg/kg) or midazolam (0.02 mg/kg). The esketamine group showed a decrease in MADRS of −15.3 (SD = 11.2), while the midazolam group had a decrease of −8.8 (SD = 9.4) at day 6, indicating a robust response to esketamine therapy. The mean difference between ketamine and midazolam was −6.5 (95% CI: −12.01, −0.99). The study exhibited a low risk of bias across all assessed domains, including the randomization process, adherence to the intended interventions, completeness of outcome data, accuracy in the measurement of outcomes, and selection of reported results.

The NCT03185819 [60] provided additional insights into the efficacy of esketamine for adolescent TRD. In this randomized controlled trial, 147 participants aged 12–18 with TRD received either esketamine (28, 56, or 84 mg) or oral midazolam (0.125 mg/kg) twice a week for four weeks alongside their current antidepressant treatment. Specifically, the midazolam group showed a reduction in CDRS-R 24 h after the initial dose of −26.2 (SD 16.72), the esketamine 28 mg group had a reduction of −29.6 (SD 18.15), the esketamine 56 mg group had a reduction of −31.8 (SD 12.92), and the esketamine 84 mg group had a reduction of −30.3 (SD 17.48). The mean difference between esketamine 28 mg and midazolam was −3.40 (95% CI: −11.28, 4.48). The mean difference between esketamine 56 mg and midazolam was −5.60 (95% CI: −11.73, 0.53). The mean difference between esketamine 84 mg and midazolam was −4.10 (95% CI: −12.35, 4.15). The study showed a low risk of bias across all evaluated domains. Randomization and allocation concealment were well-implemented, and blinding was maintained for both participants and investigators. The study adhered to its protocol, reported complete outcome data, and avoided selective reporting.

Dwyer’s crossover study [58] has shown that a single infusion of ketamine (0.5 mg/kg) significantly reduces MADRS scores compared to midazolam (0.045 mg/kg) over a 14-day period, with notable differences on days 1, 5, 6, 10, and 14. The study included 17 participants aged 13–17 with TRD, who received an infusion of either ketamine or midazolam, followed by the alternate compound two weeks later. Midazolam reduced MADRS with a mean of 24.13 (SD = 12.08), while esketamine reduced it with a mean of 15.44 (SD = 10.07). The mean difference was −8.69 (95% CI = −16.72, −0.65). The study highlighted that ketamine significantly reduced depressive symptoms 24 h after infusion compared to midazolam, and maintained efficacy up to 14 days post-infusion [58]. Additionally, 76% of participants responded to ketamine within the first 3 days post-infusion compared to 35% for midazolam, emphasizing ketamine’s rapid and sustained antidepressant effects. This study demonstrated a low risk of bias across all domains, including randomization, adherence to interventions, completeness of outcome data, outcome measurement, and selective reporting. However, we must note the small sample size, which may limit generalizability, and possible carryover effects due to the crossover design.

Cullen’s study [59] explored the efficacy and tolerability of intravenous ketamine in adolescents with TRD. Thirteen participants aged 12–18 years, who had failed to respond to two previous antidepressant trials, were administered six ketamine infusions (0.5 mg/kg) over two weeks. The study found an average decrease in CDRS-R scores by 42.5%, with five participants (38%) meeting the criteria for clinical response. Three responders showed a sustained remission at the 6-week follow-up, although relapse occurred within two weeks for the other two responders. Ketamine infusions were generally well-tolerated, with transient dissociative and hemodynamic symptoms reported. Higher doses were significant predictors of treatment response, suggesting a dose–response relationship that warrants further investigation to optimize dosing strategies. The open-label design introduced potential performance and detection biases due to the lack of blinding. While the study managed confounding factors and selection processes adequately, the small sample size and open-label nature impacted the bias assessment. Despite these limitations, the study provides preliminary evidence on ketamine’s efficacy and tolerability for adolescent TRD.

Potential causes of heterogeneity among the study results include differences in sample sizes (ranging from 13 to 147 participants), study designs (RCTs versus Open-Label), treatment protocols (varying dosages and frequencies of ketamine and ketamine administration), and outcome measures (different rating scales such as MADRS, CDRS-R, and C-SSRS).

The randomized controlled trials by Dwyer et al. [58]. and NCT03185819 [60], assessed using the RoB 2 tool, demonstrated a low risk of bias across all domains. Dwyer et al. [58] effectively managed randomization and allocation concealment, maintained blinding for participants and investigators, and adhered strictly to the protocol with comprehensive outcome reporting, enhancing the reliability of their findings on intravenous ketamine’s efficacy and safety. The NCT03185819 study similarly showed robust methodological rigor, with well-implemented randomization, thorough blinding, and the complete reporting of predefined outcomes, supporting the study’s conclusions on intranasal esketamine. Conversely, the open-label study by Cullen et al. [59]. assessed using the ROBINS-I tool showed a moderate risk of bias. The lack of blinding introduced potential performance and detection biases, while the small sample size and the single-arm design without a control group limited the study’s ability to attribute the observed effects solely to the intervention.

## 4. Discussion

TRD represents a significant challenge in adolescent psychiatry. Adolescents suffering from TRD face persistent depressive symptoms and an elevated risk of suicide, which is the second leading cause of death among this age group [8,10,61]. Despite the availability of various treatment options, approximately 40% of adolescents do not achieve remission with first-line treatments [13,14,15]. This highlights the critical need for effective interventions to address MDD with suicidal ideation and TRD in this vulnerable population.

The timely and accurate treatment of MDD with suicidal ideation and TRD in adolescents is imperative due to the significant developmental milestones occurring during adolescence. Early intervention can prevent the chronicity of depressive illness and mitigate the risk of developing additional psychiatric disorders [62,63,64]. Adolescents with untreated depression are more likely to suffer from depression and other mental health issues in adulthood, with subthreshold depression also contributing to an increased risk of future MDD [20,37,38]. Moreover, the absence of a timely treatment for depression is associated with long-term consequences such as poor academic performance, social isolation, and increased susceptibility to substance abuse [21]. Addressing these conditions promptly can have long-lasting positive effects on an adolescent’s mental health trajectory, fostering resilience and a healthier transition into adulthood [65].

The results from the studies by Zhou [57], Dwyer [58], and Cullen [59], and the NCT03185819 trial [60] provide compelling evidence on the efficacy of esketamine and ketamine in treating adolescents with TRD and MDD with suicidal ideation. Our analysis revealed a significant pooled effect size, affirming the treatments’ rapid antidepressant effects. We also identified common predictors of response, such as dosage variations and administration methods, and discussed potential adverse effects, emphasizing the treatments’ generally favorable safety profiles. These findings are particularly significant given the limited options and challenging nature of treating TRD in this age group.

Ketamine and esketamine have both shown rapid and significant improvements in depressive symptoms in adolescents with TRD, as well as in improving symptoms and suicidal ideation in adolescents with MDD. Ketamine has been effective in populations of adolescents who had failed to respond to traditional antidepressants [58,59]. Additionally, its action is rapid, as seen in Dwyer’s study, where a reduction in depressive symptoms was observed within 24 h [58]. Not only does it improve depressive symptoms as measured by the MADRS, but it also leads to improvements in specific anhedonic symptoms [59]. Regarding esketamine, it has shown efficacy in rapidly addressing suicidal ideation [33], with improvements in depressive symptoms as well. Notably, the NCT03185819 trial [60] underscores the potential of esketamine as a viable adjunctive treatment for TRD in adolescents, offering a non-intravenous route of administration that might be more practical and acceptable in clinical settings [60]. Delbello et al. also published the initial results from the clinical trial NCT03185819 concerning suicidal ideation, showing promising outcomes [66]. It is noteworthy that intranasal esketamine is currently the only form of esketamine approved for use in adults with TRD by major regulatory authorities such as the FDA and EMA. Furthermore, Lineham et al. found that adolescents with TRD who had undergone fewer prior trials of antidepressant medications and augmentation strategies responded better to ketamine [67].

In addition to its direct antidepressant effects, ketamine and esketamine have been shown to influence other depressive symptoms, often considered residual and difficult to treat, including cognitive symptoms and anhedonia [68,69]. Lan et al. assessed the short-term cognitive effects of repeated-dose ketamine in adolescents with MDD and suicidal ideation, demonstrating improvements in cognitive performance post-treatment [56], while Cullen et al. demonstrated ketamine’s efficacy on anhedonic symptoms [59]. Additionally, Lan et al. specifically assessed the efficacy of esketamine in adolescents with anxious versus non-anxious depression [55]. This study found that esketamine significantly reduced depressive symptoms and anxiety levels, demonstrating its broad applicability in adolescent mental health.

Moreover, ketamine has shown potential in specific subpopulations of adolescents with depression. Zarrinnegar et al. reported that a single dose of intravenous ketamine led to a rapid and sustained improvement in a teenager with psychotic depression [70]. Easterly and Taylor found that combining ketamine with electroconvulsive therapy (ECT) improved mood, reduced suicidal thoughts, and stabilized eating behaviors in an adolescent with multiple comorbid conditions [71]. Similarly, Jerath et al. observed significant improvements in depressive symptoms using ketamine and transcranial magnetic stimulation (TMS) in a treatment-resistant adolescent [72]. Wolfson et al. demonstrated that ketamine-assisted psychotherapy (KAP) resulted in symptomatic improvement across various psychiatric diagnoses, suggesting that integrating psychotherapy with ketamine’s rapid antidepressant effects can be beneficial [73]. These studies highlight ketamine’s potential as a versatile and effective treatment for severe depression in adolescents.

The safety and tolerability of ketamine and esketamine in adolescents with depression have been thoroughly examined. For instance, Cullen et al. found that ketamine infusions were generally well-tolerated, with only transient dissociative and hemodynamic changes [59]. Similarly, Dwyer et al. reported significant transient dissociative effects and no serious adverse events [58]. Esketamine also showed a favorable safety profile, with only mild and transient side effects [57,60]. A systematic review by Di Vincenzo et al., which included 13 studies, confirmed that ketamine appears to be safe and well-tolerated in adolescents [74]. The review highlighted that, while ketamine can produce transient dissociative symptoms, these effects are typically self-limited and resolve without intervention. Furthermore, no serious adverse events were reported across the studies included in this review.

The efficacy and rapid action of these innovative antidepressants, ketamine and its S-enantiomer esketamine, as confirmed by a few trials on adolescents, are promising. However, further studies are needed to fully understand their long-term effects and optimal usage in this population. Specifically, expanding research on the use of esketamine, which is already approved for adult TRD in an intranasal formulation, in adolescents would be beneficial. This expansion is critical because addressing TRD or MDD with suicidal ideation in adolescents promptly could prevent not only of the leading causes of death among young people, but also the exacerbation of psychiatric pathology during a crucial and formative phase of life.

## 5. Limitations and Future Perspectives

This systematic review has some limitations. Firstly, there are very few clinical studies on the use of ketamine or esketamine in adolescents. Additionally, the studies included often had small sample sizes, limiting the generalizability of the findings, and relatively short follow-up periods, which restrict our understanding of the long-term efficacy and safety of ketamine and esketamine. The included studies varied significantly in their design, including differences in dosing regimens, modes of administration (intravenous vs. intranasal), outcome measures, and geographic diversity. Consequently, it is difficult to extrapolate generalizable information on the use of ketamine and esketamine in TRD and MDD with suicidal ideation in adolescents. Future research should address these limitations by focusing on larger, multicenter trials with greater geographic variability to enhance the generalizability of the results. Additionally, incorporating long-term follow-up periods will help to better understand the durability of the antidepressant effects and potential long-term side effects.

## Figures and Tables

**Table 1:** Included studies’ characteristics.

Author, Year	Country	Study Design	Drug	Populations	Interventions	Outcome	Results Concerning Depressive Symptoms
Zhou, 2024 [57]	China	RCT	Esketamine (intravenous)	54 participants (ages 13–18) with MDD and suicidal ideation: 27 in the ketamine group, and 27 in the midazolam group	Three infusions (day 1, day 3, and day 5) of Esketamine 0.25 mg/kg (vs. three infusions of midazolam 0.02 mg/kg	MADRS C-SSRS	Both groups had significant MADRS and C-SSRS reduction at day 6; the esketamine group had significantly lower MADRS and C-SSRS score compared with the midazolam group at day 6
NCT03185819, 2018 [60]	USA	RCT	Esketamine (intranasal)	147 participants (ages 12–18) with MDD and suicidal ideation: 84 in the esketamine group, and 63 in the midalozapm group	Esketamine (28.56 or 84 mg) or oral midalozam (0.125 mg/kg) twice a week for 4 weeks	CDRS-R	Esketamine was more effective than midazolam in reducing CDRS-R scores 24 h after the initial dose, although the differences were not statistically significant
Dwyer, 2021 [58]	USA	RCT (crossover)	Ketamine (intravenous)	17 participants (ages 13–17) with TRD	A single intravenous infusion of either ketamine (0.5 mg/kg) or midazolam (0.045 mg/kg) and the alternate compound 2 weeks later	MADRS CDRS-R	Ketamine significantly reduced MADRS scores compared to midazolam over a 14-day period, with notable differences on days 1, 5, 6, 10, and 14. Both treatments significantly improved CDRS-R scores immediately after infusion, but there were no significant differences in symptom change rates over the subsequent 14 days
Cullen, 2018 [59]	USA	Open-label study	Ketamine (intravenous)	13 participants (ages 13–18) with TRD	Six ketamine (0.5 mg/kg) infusions over the course of 2 weeks	MADRS CDRS-R BDI-II SHAPS TEPS	Significant improvements were observed in CDRS-R, MADRS, BDI-II, and CGI scores, while changes in SHAPS and TEPS were not significant

BDI-II: Beck Depression Inventory-II; C-SSRS: Columbia Suicide Severity Rating Scale; CDRS-R: Children’s Depression Rating Scale-Revised; CGI-SS-R: Clinical Global Impression of Severity of Suicidality-Revised; MADRS: Montgomery–Åsberg Depression Rating Scale; MDD: Major Depressive Disorder; RCT: Randomized Controlled Trial; SHAPS: Snaith–Hamilton Pleasure Scale; TEPS: Temporal Experience of Pleasure Scale; TRD: Treatment-Resistant Depression.

## Supplementary Materials

The following supportin information can be downloaded at: https://www.mdpi.com/article/10.3390/children11070801/s1, Figure S1: Quality evaluation of RCTs; Figure S2. Quality evaluation of non-RCTs; Table S1. GRADE Assessment.
