# Supplementary material for: A Systematic Review on Ketamine and Esketamine for Treatment-Resistant Depression and Suicidality in Adolescents: A New Hope?
Source: Children (Basel). 2024 Jun 29;11(7):801. doi: 10.3390/children11070801 (PMC11274655; doi:10.3390/children11070801)
Supplement: Supplementary file 1 [file children-11-00801-s001.zip › children-3069289-supplementary.pdf]

Supplementary Materials

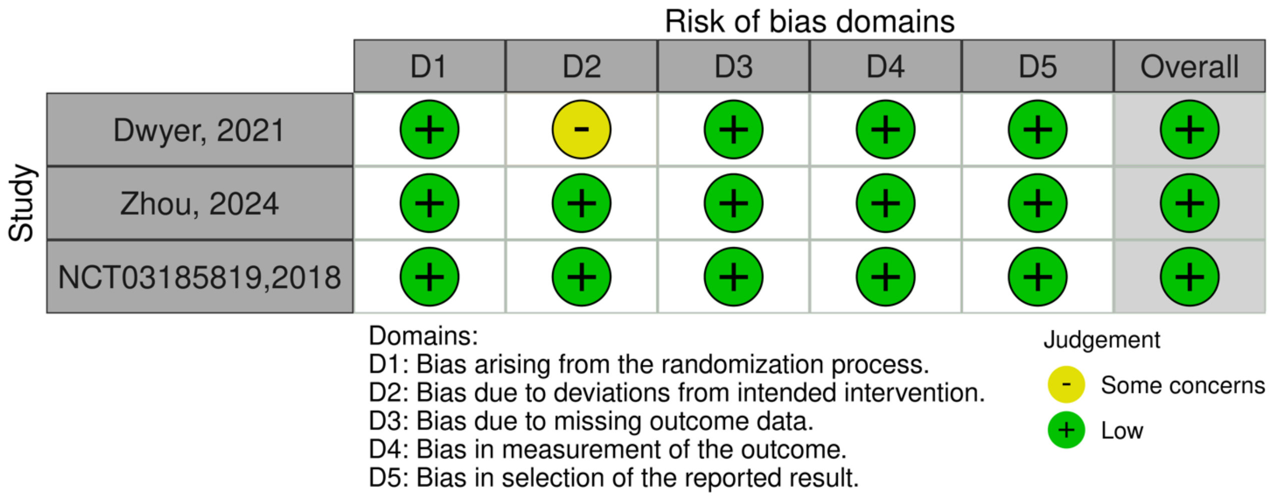

Figure S1. Quality evaluation of RCTs. Assessment based on Cochrane Risk of Bias 2.0 (RoB2) tool and displayed with Risk of bias VISualization (robvis) tool

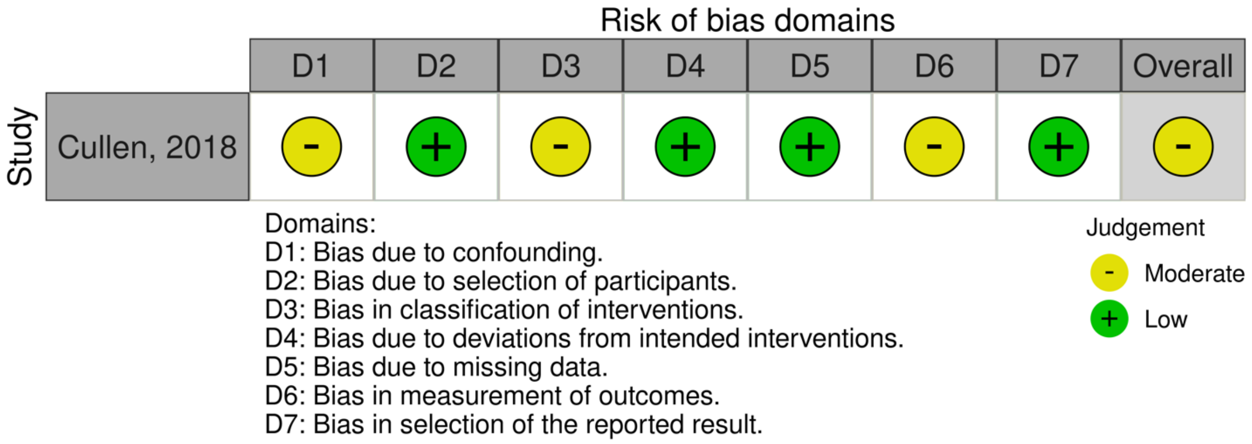

Figure S2. Quality evaluation of non-RCTs. Assessment based on Risk Of Bias In Non-randomized Studies - of Interventions (ROBINS-I) tool and displayed with Risk of bias VISualization (robvis) tool

Table S1. GRADE Assessment

Outcome	Studies	Risk of Bias	Inconsistency	Indirectness	Imprecision	Other Considerations	Overall Quality of Evidence
**Reduction in depressive symptoms**	3 RCTs, 1 open-label	Not Serious	Not Serious	Not Serious	Not Serious	None	Important
**Reduction in Suicidal Ideation**	2 RCTs,	Not Serious	Not Serious	Not Serious	Not Serious	None	Important
